# Animal Models as Tools to Investigate Antidiabetic and Anti-Inflammatory Plants

**DOI:** 10.1155/2012/142087

**Published:** 2012-07-29

**Authors:** Mohamed Eddouks, Debprasad Chattopadhyay, Naoufel Ali Zeggwagh

**Affiliations:** ^1^Moulay Ismail University, BP 21, Errachidia 52000, Morocco; ^2^ICMR Virus Unit, ID & BG Hospital, General Block 4, 57 Dr. Suresh C. Banerjee Road, Kolkata 700010, India

## Abstract

Plants have been historically used for diabetes treatment and related anti-inflammatory activity throughout the world; few of them have been validated by scientific criteria. Recently, a large diversity of animal models has been developed for better understanding the pathogenesis of diabetes mellitus and its underlying inflammatory mechanism and new drugs have been introduced in the market to treat this disease. The aim of this work is to review the available animal models of diabetes and anti-inflammatory activity along with some *in vitro* models which have been used as tools to investigate the mechanism of action of drugs with potential antidiabetic properties and related anti-inflammatory mechanism. At present, the rigorous procedures for evaluation of conventional antidiabetic medicines have rarely been applied to test raw plant materials used as traditional treatments for diabetes; and natural products, mainly derived from plants, have been tested in chemically induced diabetes model. This paper contributes to design new strategies for the development of novel antidiabetic drugs and its related inflammatory activity in order to treat this serious condition which represents a global public health problem.

## 1. Introduction

More than 1000 plants have been described as efficacious in the treatment of diabetes mellitus. However, many of these descriptions are anecdotal accounts of traditional usage, and fewer than half of these plants or plant extracts have received a thorough medical or scientific evaluation of their purported benefits. This paper reviews the preclinical *in vivo* methods and clinical procedures used to investigate the antidiabetic activity of plants and plant-derived extracts, including a consideration of the ethical issues affecting use of traditional plant treatments for diabetes [1, 2].

Animal models have been used extensively to investigate the *in vivo* efficacy, mode of action and side effects of antidiabetic plants and their active principles. Due to the heterogeneity of diabetic conditions in man, no single animal model is entirely representative of a particular type of human diabetes. Thus, many different animal models have been used, each displaying a different selection of features seen in human diabetic states [3–5]. Normal nondiabetic animals and animals with impaired glucose tolerance and insulin resistance (but not overt diabetes) have been used to demonstrate hypoglycemic activity and to investigate the mode of action of antidiabetic plant materials. It is noteworthy that agents that show a blood-glucose-lowering effect in animals are not necessarily effective in man and vice-versa. This may be due at least in part to differences in hepatic metabolism, where the metabolites are the active compounds [6–8]. Considerable variations in sensitivity to the same agent can also occur between species due to different rates of absorption, metabolism, and elimination [2, 9–11]. The most widely used animal models are small rodents, which are less expensive to maintain than larger animals and generally show a more rapid onset of their diabetic condition consistent with their short lifespan. Moreover, a greater variety of mutations leading to diabetes observed in rodents have been characterized in more detail than those in other animal groups [12–15].

## 2. *In Vivo *Animal Models of Diabetes Mellitus

### 2.1. Pharmacological Induction

Diabetes can be induced by pharmacologic, surgical, or genetic manipulations in several animal species. Most experiments in diabetes are carried out on rodents, although some studies are still performed in larger animals [16–18]. The classical model employed by Banting and Best was pancreatectomy in dogs [19–22]. It is also described prone strains to diabetes mellitus that have been employed in several researches [23–26]. Currently, the murine model is one of the most used due to the availability of over 200 well-characterized inbred strains and the ability to delete or overexpress specific genes through knockout and transgenic technologies [26–29].

Actually, till date there is neither any evidence nor any validation that a natural plant material can serve as a complete substitute for insulin. Though, several plant products have been reported to mimic the effects of insulin partially or enhance the effects of very low endogenous insulin concentrations, but none has sustained life in the total absence of insulin [19, 21, 30]. Nevertheless, animal models of insulin-dependent diabetes provide a valuable insight into the efficacy of potential adjuncts to insulin therapy in severely hypoinsulinemia states. The classifications of type 2 diabetes animal models are presented in Table 1. The main insulin-dependent models are as follows:

- spontaneous syndromes (e.g., BB rat, NOD mouse, LEW.1AR1/Ztm-iddm rat),
- experimentally induced (e.g., chemically, with alloxan or streptozotocin or surgically by near-total pancreatectomy).

These incur extensive or complete loss of pancreatic *β*-cells and the consequent lack of insulin thereby cause extreme hyperglycemia with glycosuria, polyuria, polydipsia, hyperphagia, and weight loss [31–33].

If untreated, these forms of diabetes culminate in fatal hyperosmolar ketoacidosis. The majority of studies published in the field of ethnopharmacology between 1996 and 2006 employed these models. Streptozotocin (STZ, 69%) and alloxan (31%) are by far the most frequently used drugs, and this model has been useful for the study of multiple aspects of the disease. Both drugs exert their diabetogenic action when they are administered parenterally: intravenously, intraperitoneally, or subcutaneously [34–36]. The required dose of these agents for inducing diabetes depends on the animal species, route of administration, and nutritional status. According to the administered dose of these agents, syndromes similar to either type 1, type 2 diabetes, mellitus or glucose intolerance can be induced. Protocols are available anywhere, being critical the pH and type of buffer employed as well as the preparation of the solution of either alloxan or streptozotocin in the day of the experiments [37–39]. The advantages and disadvantages of different categories of type 2 diabetic models are depicted in Table 2.

The cytotoxic action of these diabetogenic agents is mediated by reactive oxygen species (ROS), but both drugs differ in their mechanism of action. Alloxan and the product of its reduction, dialuric acid, establish a redox cycle with the formation of superoxide radicals. These radicals undergo dismutation to hydrogen peroxide with a simultaneous massive increase in cytosolic calcium concentration, which causes rapid destruction of pancreatic *β*-cells. The range of the diabetogenic dose of alloxan is quite narrow and even light overdosing may be generally toxic and may cause the loss of many animals [41–43]. This loss is likely to stem from tubular cell necrotic toxicity in kidney, in particular when too high doses of alloxan are administered. The most frequently used intravenous dose of alloxan in rats is 65 mg/kg, but when it is administered intraperitoneally (i.p.) or subcutaneously its effective dose must be higher. For instance, an intraperitoneal dose below 150 mg/kg may be insufficient for inducing diabetes in this animal species. In mice, doses vary among 100–200 mg/kg by intravenous (i.v.) route [16, 44–46].

Streptozotocin enters the pancreatic *β*-cell via a glucose transporter-GLUT2 and causes alkylation of DNA [38, 47, 48]. Furthermore, STZ induces activation of polyadenosine diphosphate ribosylation and nitric oxide (NO) release. As a result of STZ action, pancreatic *β*-cells are destroyed by necrosis. In adult rats, 60 mg/kg is the most common dose of STZ to induce insulin-dependent diabetes, but higher doses are also used. STZ is also efficacious after intraperitoneal administration of a similar or higher dose, but single doses below 40 mg/kg may be ineffective. In general, rats are considered diabetic if tail blood glucose concentrations in fed animals are greater than 200–300 mg/dL, 2 days after STZ injection [23, 49–51]. A model of type 2 diabetes can be induced in rats by either i.v. (tail vein) or i.p. treatment with STZ in the first days of life. At 8–10 weeks of age and thereafter, rats neonatally treated with STZ manifest mild basal hyperglycemia, an impaired response to the glucose tolerance test, and a loss of pancreatic *β*-cell sensitivity to glucose. It has been observed that STZ at first abolished the pancreatic *β*-cell response to glucose, but a temporary return of responsiveness appears which is followed by its permanent loss. In adult mice, STZ given in multiple low doses (40 mg/kg, i.v. for 5 days) induces insulin-dependent diabetes that is quite similar to the autoimmune forms (islet inflammation and *β*-cell death) of type 1 diabetes [26, 27, 52–54]. On the other hand, a single dose between 60 and 100 mg/kg of STZ, administered systemically, can also cause insulin-dependent diabetes, but it lacks the autoimmune profile [20, 55].

The potential problem with STZ due to its toxic effects is not restricted to pancreatic *β*-cells since it may cause renal injury, oxidative stress, inflammation, and endothelial dysfunction [34–36, 56]. The doses of alloxan and streptozotocin used in both intravenous (i.v.) and intraperitoneal (i.p.) route in different animal species are presented in Table 3.

The destruction of pancreatic *β*-cells by both drugs is associated with a huge release of insulin which makes animals more susceptible to severe hypoglycemia which may be lethal. Thus, following treatment with either STZ or alloxan, animals are fed with glucose solution (5%) for 12–24 h. Afterwards, an increase of glucose levels is observed in comparison to control animals due to insulin deficiency. It is also reported that fasted animals are more susceptible to alloxan effects and increased blood glucose in fed animals provides partial protection. In general, experimental protocols recommend that administration of either STZ or alloxan must be done in the fasting period (8–12 h) followed by addition of glucose solution to avoid hypoglycemia [43, 48, 57–59]. Besides rats, dogs, and mice, other animal species such as rabbits and monkeys have been employed to induce diabetes by these protocols, but rabbits and pigs are more resistant to STZ [16, 60–62].

In general, by using these models of diabetes induced by chemical drugs, the majority of published studies report the amount of reduction of blood glucose which is always evaluated after a period of fasting following acute or chronic treatment with a specific natural product [63–67]. Comparative studies are carried out with nondiabetic and/or diabetic animal groups treated with known antidiabetic drugs, but results do not permit to further explore the mechanism of action of the studied natural product. Glucose is measured by standard glucose oxidase or dehydrogenase assays, mainly by means of commercial meters available everywhere. Insulin determination is available in experimental animals by different methodologies (radioimmunoassay (RIA) or immunometric assays) [68–71].

It is necessary to reemphasize that natural products display several effects besides lowering blood glucose in these experimental models. In view of the lack of parallel studies of their toxicity, these models of diabetes induced by either alloxan or STZ are considered a screening step in the search for drugs for the treatment of diabetes [2, 11–13, 72].

Considering the diversity of active principles found in the crude plant extracts, some studies also include further analysis of lipids (cholesterol and triglycerides) as well as additional evaluation of the antioxidant properties of the product. This can be exemplified with studies using the fruit-pulp, seeds, and leaves extracted from *Eugenia jambolona*, a plant used in folk medicine with recognized antidiabetic properties in several countries. Despite of lack of dose standardization, plant environment selection, and toxicological studies, different other studies have confirmed that the ethanolic extracts of either the fruit-pulp or seeds of this plant have hypoglycemic, hypolipidemic, and antioxidant properties [6, 73–75]. These results suggest that numerous active principles may be present in the raw extract of the plant and highlight the need to further advance in their characterization. In line with this hypothesis, several compounds with antihyperglycemic properties have been isolated from plants. The identification of these products explains, in part, why these compounds present antioxidant, antihyperglycemic, antilipidemic properties and even enhance the process of wound healing in diabetic and nondiabetic animals. Due to the nonspecific action of compounds isolated from extracts of natural products, some studies have aggregated additional *in vitro* protocols to the *in vivo* studies, such as liver perfusion, to evaluate glucose influx inhibition, gastrointestinal absorption methodologies, and antioxidant enzyme systems, as well as liver glycogen level, among others [9, 71, 76, 77]. These protocols contribute to extend the analysis of the antidiabetic effects of certain natural products. In this context, liver perfusion methodologies with the simultaneous measurement of glucose influx help to elucidate if the natural product exerts extrapancreatic effects like metformin and glitazones. On the other hand, other studies suggest that inhibition of carbohydrate absorption may be linked to the antidiabetic properties of the natural product [10, 12]. Thus, inclusion of at least two different routes of treatment, for instance, i.p. and oral route (p.o.) can help in the analysis of the possible site of action of a studied natural product [10, 12, 69, 71, 78].

### 2.2. Surgical Models of Diabetes

Another technique used to induce diabetes is the complete removal of the pancreas. Few researchers have employed this model recently to explore effects of natural products with animal species such as rats, pigs, dogs, and primates [3, 7, 73, 79]. Limitations to this technique include high level of technical expertise and adequate surgical room environment, major surgery, high risk of animal infection, adequate postoperative analgesia and antibiotic administration, supplementation with pancreatic enzymes to prevent malabsorption, and loss of pancreatic counter regulatory response to hypoglycemia. More recently, partial pancreatectomy has been employed, but large resection (more than 80% in rats) is required to obtain mild-to-moderate hyperglycemia. In this case, small additional resection can result in significant hypoinsulinemia [9, 78, 80].

## 3. Spontaneous and Transgenic Animal Models

These models permit the evaluation of the effect of a natural product in an animal without the interference of side effects induced by chemical drugs like alloxan and STZ reported above. Several recent publications summarized the major advances in this state similar to the human condition. In some of these models, insulin resistance predominates in association with obesity, dyslipidemia, and hypertension, which provides valuable insights to study some events that are observed in human type 2 diabetes mellitus. Conversely, some strains like Ob/Ob mouse may maintain euglycemia due to a robust and persistent compensatory pancreatic *β*-cell response, matching the insulin resistance with hyperinsulinemia [4, 8, 13, 72, 75]. On the other hand, the db/db mouse rapidly develops hyperglycemia since their pancreatic *β*-cells are unable to maintain the high levels of insulin secretion required throughout life. Thus, food intake is important in determining the severity of the diabetic phenotype, and restriction of energy intake reduces both the obesity and hyperglycemia seen in this strain of mice. Another example is the spontaneously diabetic Goto-Kakizaki rat which is a genetic lean model of type 2 diabetes originating from selective breeding over many generations of glucose-intolerant nondiabetic Wistar rats. Regarding type 1 diabetes models, the NOD mouse typically presents hyperglycemia between 12 and 30 weeks of age, whereas in BB rats it occurs around 12 weeks of age. One great advantage of these models is that they can also be employed as model of atherosclerosis which represents the long-term complication of diabetes mellitus and tested against several natural products [70, 76, 78, 80–82].

Another valuable spontaneous syndrome occurs in the sand rat (*Psammomys obesus*). In the wild, these animals actively forage for a meager diet. Constrained under laboratory conditions with free access to an energy-rich diet, they become hyperphagic, obese, and hyperinsulinemic and develop insulin resistance and hyperglycemia. In later life, some of these animals incur *β*-cell failure with severe hyperglycemia. Rhesus monkeys (*Macaca mulatta*) maintained in captivity with limited space and unrestricted availability to an energy-rich diet are also prone to become obese and develop diabetes [46, 64, 83, 84]. Although this closely reflects human type 2 diabetes, primate models have received very limited use due to expense and the relatively long time period of diabetes development [36, 42, 58, 85]. The Ob/Ob mouse lacks biologically active leptin due to a mutation in the leptin gene. This model develops severe insulin resistance and marked hyperinsulinemia with gross obesity, extensive *β*-cell hyperplasia, and mild-to-moderate hyperglycemia [18, 65, 66, 84]. Thus, ob/ob mouse provides an especially challenging test for any potential treatment against insulin resistance. The Zucker fatty (fa/fa) rat, which results from a mutation in the leptin receptor, is also used as a model of insulin resistance [69, 76, 86, 87]. However, the fa/fa rat seems to exhibit more impaired glucose tolerance than overt diabetes. Cross-breeding of rats carrying the *fa* mutation has produced other insulin-resistant models which develop overt diabetes, such as the Zucker diabetic fatty (ZDF) rat and the Wistar Kyoto (WKY) fatty rat [9, 10, 14, 73, 88].

## 4. Right Model for Right Plant

The guidelines that apply to preclinical testing of a new chemical entity (NCE) do not necessarily apply to a traditional plant treatment. For example, the plant may have normal dietary ingredients traditionally taken as a raw or cooked part of the whole plant (e.g., leaf or root) or as an unrefined extract (e.g., simple decoction or infusion).

The heterogeneity of human types of diabetes and the lack of exact replicas among nonprimate animals often require efficacious studies in more than one model, especially to investigate the mode of action. Accounts of the traditional use of an antidiabetic plant in type 1 or type 2 diabetic patients provide an indication of the type of model (e.g., insulin dependent or noninsulin dependent) that might be suitable for initial investigation of hypoglycemic activity [1, 3, 78, 79, 88]. As it was noted previously, experimentally induced models of insulin-dependent diabetes are often not completely devoid of endogenous insulin. This is an important consideration when claims of an insulin substitute are being investigated. To test the efficacy of antidiabetic plants using STZ-induced or alloxan-induced diabetes in rodents, a convenient procedure is to commence plant therapy within a few days of STZ/alloxan administration before hyperglycemia becomes severe [6, 7]. Efficacy can then be judged by a slower progression and less severe hyperglycemia. If the study is continued until a parallel placebo (untreated) group develops ketoacidosis and requires insulin, this suggests that antidiabetic activity in an insulin-dependent state. However, it is possible that the therapeutic intervention has prevented complete *β*-cell destruction [6, 7]. This can be seen if insulin concentrations are measured and animals survive when the intervention is discontinued. Because some natural regeneration of islets can occur from islet remnants, long-term survival cannot be exclusively attributed to the therapeutic intervention. An alternative protocol to test efficacy in an insulin-dependent state is to introduce plant therapy to spontaneously or experimentally induced models which have already developed severe hyperglycemia and are controlled by exogenous insulin injections [71, 78, 82, 87]. When plant treatment is introduced and the dosage titrated up, evidence of reduced hyperglycemia or a reduction of insulin dosage without deterioration of glycemic control can be used as indices of efficacy [15, 73].

The selection of noninsulin-dependent models to assess efficacy of antidiabetic plants can also provide important information about mechanism of action [23, 49]. Human type 2 diabetes arises through the combined impact of insulin resistance and *β*-cell dysfunction, and it is advantageous if a model designed to test efficacy exhibits both of these pathogenic features. Therapies that ameliorate obesity and dyslipidemia offer secondary benefits to improve glycemic control; therefore, models that incorporate these features can often yield additional relevant information [29, 53]. Thus, the diabetic db/db mouse and male ZDF rat provide very useful models. It is noteworthy that a plant treatment may have no efficacy if the model in which it is tested lacks the particular pathogenic feature (e.g., insulin resistance, *β*-cell dysfunction, obesity, dyslipidemia) against which the treatment exerts its main effect. Consequently, it may be necessary to conduct studies in several animal models to establish efficacy as well as to determine mode of action [27, 30, 53, 55].

At present no drug is able to arrest the progressive loss of pancreatic *β*-cells which occurs in type 2 diabetes mellitus. According to the United Kingdom Prospective Diabetes Study (UKPDS) results, at the time of type 2 diabetes mellitus diagnoses, 50% of pancreatic *β*-cell function had already been lost [38, 42, 47, 57]. Thus, the efficacy and side effect of marketed oral antidiabetic drugs still need to be optimized. The recent introduction in the market of incretin analogs opened a new field to evaluate drugs with putative properties that may cause both proliferation and maturation of human pancreatic *β*-cells [16, 17, 83, 84]. Currently, a standard model of experimental diabetes to study effects of drugs, which could help in preventing the progressive loss of pancreatic islet function, remains to be established [76, 86, 89].

## 5. Models for Evaluation of Anti-Inflammatory Activity

Inflammation is protective and defense mechanism of the body and thus, during inflammation various pathological changes take place. The production of active inflammatory mediators is triggered by microbial products or by host proteins, such as proteins of the complement, kinins, and coagulation systems that are activated by damaged tissues. In preclinical studies, these changes can be induced by administration of the agents causing inflammation. For purpose of evaluation of anti-inflammatory activity, we will discuss some *in vivo* animal models commonly used in laboratory practice. Numerous reports have been demonstrated in increased incidence of inflammatory condition in lifestyle diseases like diabetes, as inflammation is one of the most important natural defence mechanisms. Its main purpose is to destroy the injurious agent and/or minimize its effects. Though inflammation is normally protective but, if untreated, it can go for chronic condition leading to serious complications. Inflammation is the dynamic pathological process consisting of a series of interdependent changes. Inflammation is body's response to disturbed homeostasis caused by infection, injury, trauma, and several other reasons resulting in systemic and local effects. The Roman writer Celsus in 1st century AD identified the four Cardinal Signs of inflammation as redness (Rubor), swelling or edema (Tumor), heat (Calor), and pain (Dolor) [90]. Inflammation constitutes the body's response to injury and is characterized by a series of events including the inflammatory reaction, a sensory response perceived as pain, and a repair process. The main causes of inflammatory reaction are infection (invasion and multiplication within tissues by bacteria, fungi, viruses, protozoa causing damage to the host cells), trauma penetrating injury, blunt trauma, thermal injury, chemical injury, and immunologically mediated injury (humoral or cellular), and as a result of the loss of blood supply (ischemia) [91].

Inflammation may be acute and chronic, where inflammatory response occurs in three distinct phases. The first phase is caused by an increase in vascular permeability resulting in exudation of fluids from the blood into the interstitial space, the second phase involves the infiltrations of leukocytes from the blood into the tissue and in third phase granuloma formation and tissue repair. Mediators of inflammation originate either from plasma (e.g., complement proteins, Kinins) or from cells (e.g., histamine, prostaglandins, cytokines). The production of active mediators is triggered by microbial products or host proteins, such as proteins of the complement, kinins, and coagulation systems that can cause tissue damage. Generally the mediators of inflammation are histamine, prostaglandins (PGs), leukotrienes (LTB_4_), nitric oxide (NO), platelet-activation factor (PAF), bradykinin, serotonin, lipoxins, cytokines, growth factors. Here, we will address the commonly used animal models for the evaluation of anti-inflammatory activity in laboratory along with the principle and procedure of using animal model.

## 6. Acute Inflammation 

### 6.1. Carrageenan-Induced Paw Edema in Rats [92]

This model is based on the principle of release of various inflammatory mediators by carrageenan. Edema formation due to carrageenan in the rat paw is biphasic, where in the initial phase the release of histamine and serotonin takes place. The second phase is due to the release of prostaglandins, protease and lysosome [93, 94]. Subcutaneous injection of carrageenan into the rat paw produces inflammation resulting from plasma extravasation, increased tissue water and plasma protein exudation, along with neutrophil extravasation, due to the metabolism of arachidonic acid [95]. The first phase begins immediately after injection of carrageenan and diminishes in two hours, while the second phase begins at the end of first phase and remains through three to five hours. *Procedure.* Animals are divided into three groups (*n* = 6) starved overnight with water *ad libitum* prior to the experiment. The control group receives vehicle orally, while test group receives test drug and standard drug, respectively. Left paw is marked with ink at the level of lateral malleolus; basal paw volume is measured plethysmographically by volume displacement method using plethysmometer by immersing the paw till the level of lateral malleolus. The animals are given drug treatment. One hour after dosing, the rats are challenged by a subcutaneous injection of 0.1 mL of 1% solution of carrageenan into the subplantar side of the left hind paw. The paw volume is measured again at 1, 2, 3, 4, and 5 hours after challenge. The increase in paw volume is calculated as percentage compared with the basal volume. The difference of average values between treated animals and control group is calculated for each time interval and evaluated statistically. The percent inhibition is calculated using the formula as follows: % edema inhibition = [1 − (*V*_*t*_/*V*_*c*_)] × 100 (*V*_*t*_ and *V*_*c*_ are edema volume in the drug treated and control groups, resp.).

### 6.2. Histamine-Induced Paw Edema in Rats [96]

Histamine-induced paw edema occur in earlier stage in mounting of vascular reaction in the chemically induced inflammation. Here, swelling occurs primarily due to action of histamine. Generally, histamine is released following the mast cell degranulation by a number of inflammatory mediators including interleukin-1 (IL-1). This is likely to evoke the release of neuropeptides as well as release of prostaglandins and monohydroxy eicosatetraenoic acid from endothelial cell leading to hyperalgesia and other proinflammatory effects [96]. *Procedure*: The procedure is same as that of carrageenan-induced paw edema, only instead of carrageenan the rats are challenged by a subcutaneous injection of 0.1 mL of 1% solution of histamine into the subplantar side of the left hind paw. The paw volume is measured and the percent inhibition of inflammation is calculated and compared with control group with the formula: % Inhibition = *V*_*c*_ − *V*_*t*_ × 100 (*V*_*t*_ and *V*_*c*_ are the edema volume in the drug treated and control groups, resp.).

### 6.3. Acetic-Acid-Induced Vascular Permeability [97]

The test is used to evaluate the inhibitory activity of drugs against increased vascular permeability induced by acetic acid as it can release inflammatory mediators [98]. Mediators such as histamine, prostaglandins, and leukotrienes are released following stimulation of mast cells, leading to a dilation of arterioles and venules thereby increase the vascular permeability. As a consequence, fluid and plasma protein are extravasated and edemas are formed. *Procedure.* Animals are divided into three groups (*n* = 6). The control group received vehicle orally, while other groups received test drug and standard drug, respectively, followed by the injection of 0.25 mL of 0.6% solution of acetic acid intraperitoneally. Immediately after administration, 10 mg/kg of 10% (w/v) Evan's blue is injected intravenously through the tail vain. Thirty minutes after Evan's blue injection, the animals are held by a flap of abdominal wall and the viscera irrigated with distilled water over a Petri dish. The exudate is then filtered and makes the volume up to 10 mL. The dyes leaking out into the peritoneal cavity measured spectrophotometrically using visible spectra at 10 nm and compared with the control group.

### 6.4. Xylene-Induced Ear Edema Thickness and Weight [99]

In xylene-induced ear edema model, the application of xylene induces neurogenous edema, which is partially associated with the substance P, an undecapeptide of central and peripheral nervous system, and acts as a neurotransmitter or neuromodulator in several physiological processes. Substance P is released from the neurons in the midbrain in response to stress, where it facilitates dopaminergic neurotransmission from sensory neurons in the spinal cord against noxious stimuli and excites dorsal neurons. In the periphery, release of substance P from sensory neurons causes vasodilatation and plasma extravasations suggesting its role in neurogenous inflammation. Thus, it can cause the swelling of ear in the mice. *Xylene-Induced Ear Edema (Thickness)*. The animals (mice) can be divided into five groups (*n* = 6), fasted overnight and allowed free access to water. The animals are administered with drugs to respective groups. One hour later, each animal received 30 mL of xylene using micropipette on anterior and posterior surfaces of the right ear. The left ear is considered as control. Again after one hour later, the thickness of the ear is determined using Digimatic Caliper. The percentage of ear edema is calculated based on the left ear without xylene. *Xylene-Induced Ear Edema (weight)*. The animals (mice) were divided into five groups (*n* = 6), fasted overnight and allowed free access to water. The animals are administered with drugs to respective groups. One hour later, each animal received 30 mL of xylene using micropipette on anterior and posterior surfaces of the right ear lobe. The left ear is considered as control. After one hour, the animals were sacrificed by ether anesthesia and both the ears are removed. Circular sections are taken, using a cork borer (7 mm dia), and weighed. The percentage of ear edema is calculated based on the left ear without xylene [99].

### 6.5. Arachidonic-Acid-Induced Ear Edema [100]

This model is based on the principle of metabolism of arachidonic acid by cyclooxygenase (COX) leading to the generation of PGs and thromboxanes that mediate pain and edema associated with inflammation. Inhibition of these mediators by test drug is evaluated. *Procedure.* Inflammation is induced by topical application of arachidonic acid (2 mg in 20 *μ*mL of acetone) of both surfaces of the right ear of each mouse. Rest procedure is same as that like of xylene induced ear edema (thickness and weight parameter).

### 6.6. Phorbol Myristate Acetate-Induced Ear Edema in Mice [101]

Phorbol myristate acetate (PMA) is a protein kinase C (PKC) promoter, which induces the formation of free radicals *in vivo*. It has been demonstrated that pretreatment of mouse skin by antagonists of PKC suppresses inflammation and ROS (reactive oxygen species), that involved in the synthesis of mediators and regulated the production of TNF*α*. TNF-*α* in turn stimulate PLA_2_ activity, which releases arachidonic acid from phospholipids and stimulate the activity of COX and Lipoxygenase (LOX), involved in release of different inflammatory mediators. *Procedure.* PMA (4 *μ*g per ear) in 20 *μ*L of acetone is applied to the both ear of each mouse. The left ear (control) receives the vehicle. Test drug is administered 1 h before PMA application. Two control groups are used, a group with application of PMA on the right ear and a positive control group that receive standard drug. Six hours after PMA application, the mice are killed by cervical dislocation and a 6 mm diameter disc from each ear is removed with a metal punch and weigh. Ear edema is calculated by subtracting the weight of the left ear (vehicle) from the right ear (treatment) and is expressed as a reduction in weight with respect to the control group.

### 6.7. Myeloperoxidase (MPO) Assay [102]

MPO is present in neutrophil and in monocytes and macrophages (less amount). It is known that the level of MPO activity is directly proportional to the neutrophil concentration on the inflamed tissue. Inhibition of MPO activity by the drug preventing the generation of oxidants such as hypochlorous acid. *Procedure.* Tissue samples of each ear, from the PMA model, are assessed biochemically with neutrophil marker enzyme MPO. The ear tissue is homogenized in 50 mM K_2_PO_4_ (pH 6) containing 0.5% hexadecyl trimethylammonium bromide (HTAB) using a homogenizer. After freeze-thawing thrice, the samples are centrifuged at 2500 pm for 30 min at 4°C and the resulting supernatant is assayed spectrophotometrically for MPO determination. In brief, 40 *μ*L sample is mixed with 960 *μ*L of 50 mM phosphate buffer (pH 6), containing 0.167 mg/mL O-dianisidine dihydrochloride and 0.0005% hydrogen peroxide. The change in absorbance at 460 nm is measured with spectrophotometer. MPO activity data is presented as units per mg of tissue. One unit of MPO activity is defined as that degrading 1.0 *μ*mol of peroxide per minute at 25°C.

### 6.8. Oxazolone-Induced Ear Edema in Mice [103]

The oxazolone-induced ear edema in mice is a model of delayed contact hypersensitivity that permits the quantitative evaluation of the topical and systemic anti-inflammatory activity of a compound following topical administration. *Procedure.* Mice are divided into various groups (*n* = 6), and a fresh 2% solution of oxazolone (4-ethoxymethylene-2-phenyl-2-oxazolin-5-one) in acetone is prepared. The mice are sensitized by application of halothane anesthesia (0.1 mL on the shaved abdominal skin or 0.01 mL the inside of both ears). The mice were challenged with 0.01 mL 2% oxazolone solution 8 days later, under anesthesia, inside the right ear (control) or 0.01 mL of oxazolone solution in which the test compound or standard drug is dissolved. Groups of 10 to 15 animals are treated with the irritant alone or with the solution of the test compound. The left ear remains untreated. The maximum inflammation occurs 24 h later, when the animals are sacrificed under anesthesia and a disc of 8 mm diameter is punched from both sides. The discs are immediately weighed on a balance and the weight difference is an indicator of the inflammatory edema.

## 7. Subacute Model

### 7.1. Carrageenan-Induced Granuloma Pouch Model [104]

Carrageenan-induced granuloma pouch model is an excellent subacute inflammatory model in which fluid extravasations, leukocyte migration, and various biochemical exudates involved in inflammatory response can be detected readily. The air pouch has the advantage of supplying a suitable space for the induction of inflammatory responses. The injection of irritants such as carrageenan into subcutaneous air pouch on the dorsal surface of rats initiates an inflammatory process. *Procedure.* The animals used in this method are rats divided into five groups (*n* = 6), fasted overnight, and allowed free access to water. The animals are administered with vehicle, standard drug, and test drug. One hour after dosing, the back of the animal is shaved and disinfected. With a very thin needle, subcutaneous dorsal granuloma pouch is made in ether anaesthetized rats by injecting 6 mL of air, followed by injection of 4 mL of 2% carrageenan in normal saline to avoid any leakage of air and the treatment continued for seven consecutive days. On day 8, the pouch is opened under anesthesia and the amount of exudates was collected with a syringe. The average volume of exudates, total WBC count, and weights of granuloma is determined.

### 7.2. Formalin-Induced Paw Edema [105]

This model is based upon the ability of test drug to inhibit the edema produced in the hind paw of the mice after injection of formalin. The nociceptive effect of formalin is biphasic, an early neurogenic component followed by a later tissue-mediated response. In the first phase, there is release of histamine, 5-HT, and kinin, while the second phase is related to the release of prostaglandins. *Procedure.* The animals are divided into three groups (*n* = 6), and inflammation is produced by subplantar injection of 20 mL of freshly prepared 2% formalin in the right hind paw. The paw thickness is measured by Plethysmometrically 1 h before and after formalin injection. The drug treatment is continued for 6 consecutive days. The increase in paw thickness and percentage inhibition are calculated and compared with control group.

## 8. Chronic Model

### 8.1. Cotton Pellet-Induced Granuloma in Rats [106]

This model is based on the foreign body granuloma that can provoke by subcutaneous implantation of pellets of compressed cotton in rats. After several days, giant cells and undifferentiated connective tissue can be observed beside the fluid infiltration. The amount of newly formed connective tissue can be measured by weighing the dried pellets after removal. More intensive granuloma formation has been observed if the cotton pellets have been impregnated with carrageenan. *Procedure.* The rats are divided into five groups (*n* = 6), fasted overnight, and allowed free access to water. The animals are administered with vehicle, standard drug and test drugs. One hour after the first dosing, the animals are anesthetized with anesthetic ether and 20 mg of the sterile cotton pellet is inserted one in each axilla and groin of rats by making small subcutaneous incision. The incisions are sutured by sterile catgut [106] and the animals are sacrificed by excess anesthesia on 8th day and cotton pellets are removed surgically. Pellets are separated from extraneous tissue and dried at 60°C until weight become constant. The net dry weight, that is after subtracting the initial weight of the cotton pellet will be determined. The average weight of the pellet of the control group as well as of the test groups is calculated. The percent change of the granuloma weight relatively with vehicle control is determined and statistically evaluated, using the formula: % inhibition = *W*_*c*_ − *W*_*d*_/*W*_*c*_ × 100. *W*_*d*_ = difference in pellet weight of the drug treated group; *W*_*c*_ = difference in pellet weight of the control group.

### 8.2. The Glass Rod Granuloma [107]

The glass rod granuloma, described by Vogel, reflects the chronic proliferative inflammation. Of the newly formed connective tissue not only wet and dry weight, but also chemical composition and mechanical properties can be measured. *Procedure.* Glass rods (6 mm dia, 40 mm length) with rounded ends can be made in flame. Rods are sterilized before implantation. Male Sprague-Dawley rats (130 g) are anaesthetized, the back skin shaved and disinfected. From an incision in the caudal region, a subcutaneous tunnel is formed in cranial direction with a closed blunted forceps. One glass rod is introduced into this tunnel finally lying on the back of the animal. The incision wound is closed by sutures. The animals are kept in separate cages. The rods remain in situ for 20 or 40 days. Treatment with drugs is either during the whole period or only during the last 10 or 2 days. At the end, the animals are sacrificed under CO_2_ anesthesia. The glass rods are prepared together with the surrounding connective tissue which forms a tube around the glass rod. By incision at one end, the glass rod is extracted and the granuloma sac inverted forming a plain piece of pure connective tissue. Wet weight of the granuloma tissue is recorded and the specimens are kept in a humid chamber until further analysis. Finally, the granuloma tissue is dried and the dry weight is recorded.

## 9. Estimation of Proinflammatory Mediators and Cytokines *In Vitro*

### 9.1. Lipopolysaccharide Stimulation of THP-1 Cells

The human monocytic leukemia cell line THP-1 was used for studying antiinflammatory potential of natural products or pharmaceuticals as it is a highly differentiated monocytic cell line with phagocytic properties and has Fc as well as C3b receptors. Indeed, it is the most commonly used model to study the biology of foam cell formation because it can be easily induced to a macrophage phenotype after phorbol ester treatment [108]. Furthermore, these cells have been reported to produce proinflammatory cytokines (IL-1, IL-6, and TNF) and chemokines (IL-8 and MCP-1) in response to lipopolysaccharide (LPS) stimulation [109, 110]. The THP-1 cell line, rather than human monocytes, was used as *in vitro* model system to minimize variability and to allow for high throughput. Using the THP-1 cells, one can demonstrated that, under hyperglycemic conditions, superoxide anion and IL-6 release are increased, as observed in diabetic monocytes, and can elucidated the molecular mechanisms that mediate the increased superoxide anion and cytokine release from diabetic monocytes [111–113]. Thus, the THP-1 cell line is the best *in vitro* model system to understand monocyte/macrophage biology as it relates to human disease.

The cells will be grown in 75 mm^2^ flasks in RPMI supplemented with fetal bovine serum until they attained 70% confluency. On reaching confluency, the cells will be plated in 12-well tissue culture plates (5 × 10^5^ cells/mL) in serum-free medium at 37°C in 5% CO_2_. The cells will be challenged with different concentrations (0–1000 *μ*g/L) of LPS for different times (4, 12, and 24 h). The supernatants will be harvested after each time point and stored frozen at −20°C until analysis. IL-1*β*, TNF-*α*, and IL-6 will be quantified in all supernatants. The cells were lysed in 0.1 mol/L NaOH and the results for release of each cytokine will be reported as per milligram of protein. The intra- and interassay CVs for the cytokine assays will be <10%, and the specific time points at which maximum cytokine stimulation achieved will be noted.

### 9.2. Testing the Antiinflammatory Activity of Various Compounds

The various compounds including dietary supplements and pharmacologic agents can be tested on this model system. The TNF-*α* was the cytokine released at the earliest time point (4 h as opposed to 24 h for IL-1*β* and IL-6) and also at the lowest LPS concentration (half maximum 50 *μ*g/L). Therefore, testing the antiinflammatory effects of such compounds, THP-1 cells will be incubated with LPS (50 *μ*g/L) for a duration of 4 h and TNF-*α* concentrations will be assayed in supernatants. THP-1 cells will be pretreated with different concentrations of various compounds at biologically relevant concentrations. After 1 h of pretreatment with these compounds, the cells will be challenged with LPS (50 *μ*g/L) for 4 h. The supernatants will be used for measurement of TNF-*α*, at the range of calibrators of 0–1000 ng/L.

### 9.3. Cell Viability Assay (MTT Assay)

Cytotoxicity studies can be performed in 96-well plates. The mechanically scraped THP-1 cells will be plated at 1 × 10^5^/well in 96-well plates containing 100 *μ*L of DMEM with 10% heat-inactivated FBS and incubated for overnight. Test samples will be dissolved in DMSO (at 0.1% concentrations). After overnight incubation, the test material will be added, and the plates will be incubated for 24 h. Cells will be washed once and added with 50 *μ*L of FBS-free medium containing MTT (5 mg/mL). After 4 h of incubation at 37°C, the medium will be discarded and the formazan blue that formed in the cells will be dissolved in DMSO (100 *μ*L), and the optical density will be measured at 540 nm.

### 9.4. Assay of Proinflammatory Mediators and Cytokines

*Nitrite Assay*. Nitrite (NO) accumulation can be estimated as a measure of NO production, using the Griess reaction. Briefly, 100 *μ*L of cell culture medium will be mixed with 100 *μ*L of Griess reagent (equal volumes of 1% (w/v) sulfanilamide in 5% (v/v) phosphoric acid and 0.1% (w/v) naphthylethylenediamine-HCl), incubated at room temperature for 10 min. Absorbance at 550 nm will be measured by a microplate reader, using fresh culture medium as a blank for all experiments. The amount of nitrite in samples will be measured by standard curve prepared by using sodium nitrite solutions. *PGE2, TNF*-*α, Interleukine (IL-2, IL-6, etc.) Assay*. Prostaglandin E2 (PGE2), tumor necrosis factor *α* (TNF-*α*) interleukine levels in macrophage culture medium will be quantified by commercially available enzyme immunoassay (EIA) kits according to the manufacture's instructions.

### 9.5. Western Blot Assay of Cellular Proteins

Cellular proteins extracted from control and drug treated THP-1 cells can also be measured. The washed cell pellets will be resuspended in extraction lysis buffer (50 mM HEPES pH 7.0, 250 mM NaCl, 5 mM EDTA, 0.1% Nonidet P-40, 1 mM phenylmethylsulfonyl fluoride, 0.5 mM dithiothreitol, 5 mM NaF, and 0.5 mM Na orthovanadate) containing 5 *μ*g/mL each of leupeptin and aprotinin and incubated with 15 min at 4°C. After removal of cell debris by microcentrifugation, supernatants can quickly freeze. Protein concentration will be determined by protein assay reagent according to the manufacture's instruction. 40–50 *μ*g of cellular proteins from treated and untreated cell extracts will be electroblotted onto nitrocellulose membrane following separation on a 10% sodium dodecyl sulfate (SDS)-polyacrylamide gel electrophoresis. The immunoblot will be incubated overnight with blocking solution (5% skim milk) at 4°C, followed by incubation for 4 h with a 1 : 500 dilution of monoclonal anti-iNOS and COX-2 antibody (Santacruz, CA, USA). Blots will be washed twice with PBS and incubated with a 1 : 1000 dilution of horseradish peroxidase-conjugated goat anti-mouse IgG secondary antibody (Santacruz, CA, USA) for 1 h at room temperature. Blots will be rewashed thrice in Tween 20/Tris buffered saline (TTBS) and then developed by enhanced chemiluminescence (Amersham Life Science, Arlington Heights, IL, USA).

## 10. Conclusion

The above models have broad spectrum for evaluation of antidiabetic and related anti-inflammatory activity. In spite of the worldwide use of herbs and medicinal plants, the effective treatment of diabetes with phytochemicals has not been validated with scientific criteria which may support their substitution for the current therapy. Although some studies have been published with raw natural products, they have not shed light on the mechanisms of action of these products. This implies that several models are necessary to demonstrate that a putative natural product exerts antihyperglycemic activity. Thus, by focusing on other targets of pancreatic islet cell dysfunction, new models may help to elucidate effects of medicinal plants employed in the treatment of diabetes mellitus.

In different models, the inflammation has produced by different inducers by releasing inflammatory mediators. Each is having different mechanism of action for producing inflammation either by increased in vascular permeability, infiltrations of leukocytes or granuloma formation and tissue repair. Among these methods, the most commonly employed techniques is based upon the ability of such agents to inhibit the edema produced in the hind paw of rat after injection of a phlogistic agent (irritant) like brewer's yeast, formaldehyde, dextran, egg albumin, kaolin, aerosil, sulfated polysaccharides (carrageenan or naphthoylheparamine), as well as histamine, xylene, arachidonic acid, phorbol myristate acetate, oxozolone, croton oil, and formalin. For evaluating the most effective and widely used model for inflammation is carrageenan-induced paw edema, carrageenan is polysaccharides of sulfated galactose units and is derived from Irish Sea moss *Chondrous crispus*, which initially releases histamine and serotonin followed by prostaglandins, protease, and lysosomes. In Histamine-induced paw edema, histamine causes vasodilation and increases in vascular permeability followed by edema. Xylenereleases substance P from sensory neurons cause's vasodilatation and plasma extravasations. While arachidonic acid administration produces metabolism of arachidonic acid by cyclooxygenase leading to the generation of PGs and thromboxanes, that mediate pain and edema. Phorbol myristate acetate synthesizes mediators and regulates the production of TNF*α* which in turn stimulate PLA_2_ activity. In acetic acid induced vascular permeability, acetic acid causes dilation of arterioles and venules and increased vascular permeability by releasing histamine, prostaglandins, and leukotrienes following stimulation of mast cells and myeloperoxidase in neutrophils indicates intensity of inflammation. In air pouch model formation of exudates with migration of leukocytes and interleukins takes place, due to angiogenesis, nitric oxide synthesis, and Kinin release. Angiogenesis in a chronic inflammatory state, which facilitates migration of inflammatory cells to the inflammatory site and supplies nutrients and oxygen to granulation tissue. Thus, the suppression of angiogenesis in granulation tissue is important to suppress the development of chronic granulation tissue [114]. Nitric oxide synthesis (NO), by inducible nitric oxide synthase (iNOS), increases in inflammation and leads to cellular injury; while kinins cause vasodilatation, increase vascular permeability and WBC migration in the early stages of the inflammation, and are also responsible for, collagen formation in the later stage. It may also be responsible for the vascular flushing that occurs in the carcinoid syndrome and also implicated in the endotoxin shock, hereditary angioneurotic edema, anaphylaxis, arthritis, and in acute pancreatitis. Kinins degranulate mast cells to release histamine and other mediators and plasma extravasation by contraction of vascular endothelial cells. Kinins are potent algogenic substances, which induce pain by directly stimulating nociceptors in skin joint, and muscles [96]. In cotton-pellet-induced or glass rod granuloma, the foreign (cotton or glass rod) implanted in the skin is producing undifferentiated connective tissue indicating inflammation. The amount of newly formed connective tissue is measured by weighing the dried pellet after removal as an index of the extended severity of inflammation and thus, which indicates the proliferative phase of the inflammation of microphages, neutrophils, fibroblasts and collagen formation. Therefore, a decrease in the granuloma formation indicates the suppression of the proliferative phase [115].

## Figures and Tables

**Table 1:** Classification of type 2 diabetes models in animals.

Model category	Type 2 diabetic models	
	Obese	Nonobese
(I) Spontaneous or genetically derived diabetic animals	*ob/ob* mouse, *db/db* mouse	Cohen diabetic rat, GK rat Torri rat Nonobese C57BL/6 (Akita) mutant mouse, ALS/Lt mouse
	KK mouse, KK/A_y_ mouse	
	NZO mouse	
	NONcNZO10 mouse	
	TSOD mouse, M16 mouse	
	Zucker fatty rat, ZDF rat	
	SHR/N-cp rat, JCR/LA-cp rat	
	OLETF rat	
	Obese rhesus monkey	

(II) Diet/nutrition induced diabetic animals	Sand rat C57/BL 6J mouse, Spiny mouse	—

(III) Chemically induced diabetic animals	GTG-treated obese mice	Low-dose ALX or STZ adult rats, mice, and so forth; Neonatal STZ rat

(IV) Surgical diabetic animals	VMH lesioned dietary obese diabetic rat	Partial pancreatectomized animals, for example, dog, primate, pig, and rat

(V) Transgenic/knock-out diabetic animals	*β*_ 3_ receptor knockout mouse	Transgenic or knockout mice involving genes of insulin, insulin receptor, and its components of downstream
	Uncoupling protein (UCP1) knock-out mouse	Insulin signaling, for example, IRS-1, IRS-2, GLUT-4, PTP-1B, and others
		PPAR-g tissue-specific knockout mouse
		Glucokinase or GLUT 2 knockout mice
		Human islet amyloid polypeptide (HIP) over expressed rat

KK: Kuo Kondo; KK/A_y_: yellow KK obese; VMH: ventromedial hypothalamus; ZDF: Zucker diabetic fatty; NZO: New Zealand obese; TSOD: Tsumara Suzuki obese diabetes; SHR/N-cp: spontaneously hypertensive rat/NIH-corpulent; JCR: James C Russel; OLETF: Otuska Long Evans Tokushima fatty; GTG: gold thioglucose; ALX: alloxan; STZ: streptozotocin; GLUT: glucose transporter; IRS: insulin receptor substrate; GK: Goto-Kakizaki; PPAR: peroxisome proliferator activated receptor; PTP: phosphotyrosine phosphatase; ALS: alloxan sensitive.

**Table 2:** Advantages and disadvantages of different categories of type 2 diabetic animal models*.

Model category	Advantages	Disadvantages
(I) Spontaneous diabetic animals	Development of type 2 diabetes is of spontaneous origin involving genetic factors, and the features resemble human type 2 diabetes	Highly inbred, homogenous and mostly monogenic. Inheritance and development is genetically determined, unlike heterogeneity of humans
	Most inbred animal models are homogeneous and environmentally controlled, that allows easy genetic dissection	Limited availability and expensive. Mortality due to ketosis is high in animals with brittle pancreas (db/db, ZDF rat* P. obesus*, etc.), and it requires insulin in later stage for survival
	Variability of results is minimum and require small sample size	Require sophisticated maintenance

(II) Diet/nutrition induced diabetics	Develop diabetes with obesity due to over nutrition like diabesity syndrome of human	Mostly require long dietary treatment
	Toxicity of chemicals on other vital organs can be avoided	No frank hyperglycaemia develops upon dietary treatment in normal animals and hence unsuitable for screening antidiabetic agents on circulating glucose parameters

(III) Chemical induced diabetic animals	Selective loss of pancreatic beta cells (alloxan/STZ) leaving alpha and delta cells intact	Hyperglycaemia develops by cytotoxic action on the beta cells, leads to insulin deficiency rather than insulin resistance
	Residual insulin secretion help animals to live long without insulin treatment	Diabetes induced by chemicals is less stable and is reversible due to spontaneous regeneration of beta cells. Thus, care is required to assess beta cell function in long-term experiments
	Ketosis and mortality is relatively less	Chemically induced toxicity on other organs along with its cytotoxic action on beta cells
	Comparatively cheaper, easier to develop and maintain	Variability of results on development of hyperglycaemia is high

(IV) Surgical diabetic animals	Avoids cytotoxic effects of chemical diabetogenes on other organes	Involvement of cumbersome technical and post operative procedures
	Resembles human type 2 diabetes due to reduced islet beta cell mass	Occurrence of some digestive problems, due to excision of exocrine portion leads to the deficiency of amylase
		Dissection of alpha cells (secreting glucagon) along with beta cells leads to the counter regulatory response to hypoglycaemia
		Mortality is comparatively higher

(V) Transgenic/knock out diabetic animals	*In vivo* effect of single gene or mutation on diabetes can be investigated	Highly sophisticated and costly for production and maintenance
	Dissection of complex genetics of type 2 diabetes is easier	Expensive for regular screening experiments

*After [40].

**Table 3:** The doses of two chemical diabetogenes in different species.

Chemicals	Species	Dose (s) in mg/kg
Alloxan	Rat	40–200 (i.v./i.p.)
	Mice	50–200 (i.v./i.p.)
	Rabbit	100–150 (i.v.)
	Dog	50–75 (i.v.)

Streptozotocin	Rat	35–65 (i.v./i.p.)
	Mice	100–200 (i.v./i.p.)
	Hamster	50 (i.p.)
	Dog	20–30 (i.v.)
	Pig	100–150 (i.v.)
	Primates	50–150 (i.v.)

i.v.: intravenous; i.p.: intraperitoneal.

After [40].
